# Approximate Solution of Time-Fractional Advection-Dispersion Equation via Fractional Variational Iteration Method

**DOI:** 10.1155/2014/769713

**Published:** 2014-01-22

**Authors:** Birol İbiş, Mustafa Bayram

**Affiliations:** ^1^Department of Basic Sciences, Turkish Air Force Academy, Istanbul, Turkey; ^2^Department of Mathematical Engineering, Faculty of Chemical and Metallurgical Engineering, Yıldız Technical University, Istanbul, Turkey

## Abstract

This paper aims to obtain the approximate solution of time-fractional advection-dispersion equation (FADE) involving Jumarie's modification of Riemann-Liouville derivative by the fractional variational iteration method (FVIM). FVIM provides an analytical approximate solution in the form of a convergent series. Some examples are given and the results indicate that the FVIM is of high accuracy, more efficient, and more convenient for solving time FADEs.

## 1. Introduction

Many problems in mechanical engineering, physics, biology, chemistry, control theory, fluid mechanics, signal processing, viscoelasticity, electromagnetism, electrochemistry, thermal engineering, and many other physical processes are modeled by fractional differential equations (FDEs) or fractional partial differential equations (FPDEs) [1–9]. The solution of FDEs has been recently studied. In most cases, FDEs do not have analytical solutions, so these equations have been solved by using various analytical and numerical methods. The time-fractional advection-dispersion equations, which are a special type of FPDEs, have been applied to many problems [10–18]. There may be several methods for solving FADEs such as variable transformation [19], finite element method [20], Adomian decomposition method (ADM) [21], implicit and explicit difference method [22], homotopy analysis method (HAM) [23], optimal homotopy asymptotic method [24], homotopy perturbation method (HPM) [25], Green function [26], and least-squares spectral method [27].

In this study, the following time FADE with the initial condition is discussed:

(1) ∂αu(x,t)∂tα=κ(x,t)∂2u(x,t)∂x2+ν(x,t)∂u(x,t)∂x+g(x,t),                     x>0,  t>0,  0<α≤1,u(x,0)=u0(x),

where (*x*, *t*), *κ*(*x*, *t*), and *ν*(*x*, *t*) represent the solute concentration, the dispersion coefficient, and the average fluid velocity, respectively.

## 2. Preliminaries

Some necessary definitions, lemmas, and properties of the fractional calculus are reviewed in this section [28, 29].

**Definition 1**

The Riemann-Liouville fractional integral of order *α* is defined as

(2)   0Ixαu(x)=1Γ(α)∫0x(x−ξ)α−1u(ξ)dξ, x>0,

where *α* ≥ 0, *f* ∈ *C*_*μ*_, *μ* ≥ −1.

**Definition 2**

The modified Riemann-Liouville fractional derivative of order *α* is defined as

(3) D0xαf(x)=1Γ(n−α)dndxn∫0x(x−ξ)n−α(f(ξ)−f(0))dξ,              0≤x≤1,  n∈Z+,  n−1≤α<n.

The properties of the modified Riemann-Liouville fractional derivative are

(i) product rule for fractional derivatives:
  (4) D0xα(uv)=(D0xαu)v+u(D0xαv),
(ii) fractional Leibniz formula:
  (5) Ixα0D0xαu(x)=u(x)−u(0), 0<α≤1,
(iii) integration by parts for fractional order:
  (6) Iabα[(Dxαu)v]=(uv)|ab−Iabα[u(Dxαv)].

**Definition 3**

Limit form of the fractional derivative is defined as

(7) u(α)=lim⁡h→0Δα[u(x)−u(0)]hα.

**Definition 4**

Fractional derivative is defined for compounded functions as follows:

(8) dαu≅Γ(1+α)du, 0<α<1.

**Definition 5**

The integral with respect to (*dx*)^*α*^ is defined as

(9) du≅u(x)(dx)α, x≥0,  u(0)=0,  0<α<1.

**Definition 6**

The following equality is provided for the continuous function *u* : *R* → *R* and *x* → *u*(*x*) has a fractional derivative of order *kα* (*k* ∈ *Z* ^+^ and 0 < *α* ≤ 1):

(10) u(x+h)=∑k=0∞hαk(αk)!uαk(x),

where *u* ^(*αk*)^ is the derivative of order *kα* of *u*(*x*).

When substituting  *h* → *x* and *x* → 0 in (10), we get the fractional McLaurin series:

(11) u(x)=∑k=0∞xαk(αk)!uαk(0).

**Lemma 7**

The solution of continuous function *f*(*x*) in (8) is

(12) u=∫0xu(ξ)(dξ)α=α∫0x(x−ξ)α−1u(ξ)dξ, 0<α≤1.

For example, when (12) is applied for function *u*(*x*) = *x* ^*β*^, one gets

(13) ∫0xu(ξ)(dξ)α=∫0xξβ(dξ)α=Γ(α+1)Γ(β+1)Γ(α+β+1)xα+β,                        0<x≤1.

## 3. Fractional Variation Iteration Method (FVIM) 

Equation (1) with initial conditions is considered to describe the solution procedure of the FVIM. Using the VIM developed by He [30], a correction function for (1) can be set as follows:

(14) un+1(x,t)=un(x,t) +Iα[λ(x,t)(∂αun(x,t)∂tα−ν(x,t)∂un(x,t)∂x−κ(x,t)∂2un(x,t)∂x2−g(x,t))],

(15) un+1(x,t)=un(x,t) +1Γ(α)∫0t(t−ξ)α−1λ(x,ξ)×(∂αun(x,ξ)∂ξα−ν(x,ξ)∂un(x,ξ)∂x−κ(x,ξ)∂2un(x,ξ)∂x2−g(x,ξ))dξ.

New correction functional is obtained as follows by combining (12) and (15):

(16) un+1(x,t)=un(x,t) +1Γ(α+1)∫0tλ(x,ξ)×(∂αun(x,ξ)∂ξα−ν(x,ξ)∂un(x,ξ)∂x−κ(x,ξ)∂2un(x,ξ)∂x2−g(x,ξ))(dξ)α,

where *λ* is a Lagrange multiplier which can be determined optimally through the variational theory. Here ${\left({\tilde{u}}_{n}\right)}_{x}$ and ${\left({\tilde{u}}_{n}\right)}_{xx}$ are considered as restricted variations; that is, $\delta {\tilde{u}}_{n}=0$. Making the above functional stationary,

(17) δun+1(x,t)=δun(x,t)+δΓ(α+1)∫otλ(x,ξ)×(∂αun(x,ξ)∂ξα−ν(x,ξ)∂u~n(x,ξ)∂x−κ(x,ξ)∂2u~n(x,ξ)∂x2−g(x,ξ))(dξ)α,δun+1(x,t)=δun(x,t)+λδun(x,ξ)|ξ=t −1Γ(α+1)∫0t∂αλ(x,ξ)∂ξαδun(x,ξ)(dξ)α,

with the property from (4) and (6), *λ*(*x*, *ξ*) must satisfy

(18) ∂αλ(x,ξ)∂ξα=0,  1+λ(x,ξ)|ξ=t=0.

Therefore, *λ*(*x*, *ξ*) is determined as

(19) λ(x,ξ)=−1.

Substituting (19) into the functional (16) gives the iteration formulation as

(20) un+1(x,t)=un(x,t) −1Γ(α+1)∫0t(∂αun(x,ξ)∂ξα−ν(x,ξ)∂un(x,ξ)∂x−κ(x,ξ)∂2un(x,ξ)∂x2−g(x,ξ))(dξ)α.

We start by selecting an appropriate initial function *u*_0_(*x*, *t*); the consecutive approximations *u*_*n*_(*x*, *t*) of *u*(*x*, *t*) can be easily achieved. Generally, the initial values are chosen as zeroth approximation *u*_0_(*x*, *t*). Consequently, the solution *u*(*x*, *t*) of (1) is obtained by (*x*, *t*) = lim⁡_*n*→*∞*_⁡*u*_*n*_(*x*, *t*).

## 4. Approximate Solutions of Time FADEs

In this section, in order to show the applicability and efficiency of the FVIM for solving time FADEs, some illustrative examples are given.

**Example 8**

Firstly, the following time FADE subject to the initial condition is considered [24]:

(21) ∂αu(x,t)∂tα=μ∂2u(x,t)  ∂x2−∂u(x,t)∂x, t>0,  0<α≤1,u(x,0)=e−x.

The corresponding iterative formula (20) for (21) can be derived as

(22) un+1(x,t)=un(x,t) −1Γ(α+1)∫0t(∂αun(x,ξ)∂ξα+∂un(x,ξ)∂x  −μ∂2un(x,ξ)∂x2)(dξ)α.

Starting with *u*_0_(*x*, *t*) = *u*(*x*, 0) = *e* ^−*x*^, by the iterative formula (22), we derive the following results:

(23) u1(x,t)=e−x−1Γ(α+1)∫0t(∂αu0(x,ξ)∂ξα+∂u0(x,ξ)∂x  −μ∂2u0(x,ξ)∂x2)(dξ)α=e−x+e−x(1+μ)  Γ(α+1)∫0t(dξ)α=e−x[1+(1+μ)Γ(α+1)tα],u2(x,t)=e−x[1+(1+μ)  Γ(α+1)tα+(1+μ)2  Γ(2α+1)t2α],u3(x,t)=e−x[1+(1+μ)Γ(α+1)tα+(1+μ)2Γ(2α+1)t2α+(1+μ)3Γ(3α+1)t3α]⋮

Consequently, the approximate solution is obtained as follows:

(24) u(x,t)=lim⁡n→∞un(x,t)=lim⁡n→∞∑k=0ne−x(1+μ)kΓ(kα+1)tkα=e−xEα((1+μ)tα),

where *E*_*α*_((1 + *μ*)*t* ^*α*^) is the Mittag-Leffler function.

**Example 9**

Now, the following time FADE subject to the initial condition is considered [21]:

(25) ∂αu(x,t)∂tα=κ∂2u(x,t)∂x2−ν∂u(x,t)∂x, t>0,  0<α≤1,u(x,0)=sin(x).

The corresponding iterative formula (20) for (25) can be derived as

(26) un+1(x,t)=un(x,t) −1Γ(α+1)∫0t(∂αun(x,ξ)∂ξα+ν∂un(x,ξ)∂x−κ∂2un(x,ξ)∂x2)(dξ)α.

Starting with *u*_0_(*x*, *t*) = *u*(*x*, 0) = *x* ^2^, by formula (26), we derive the following results:

(27)  u1(x,t)=sin(x)−(νcos⁡(x)+κsin(x))tαΓ(α+1), u2(x,t)=sin(x)−(νcos⁡(x)+κsin(x))tαΓ(α+1)+(2κνcos⁡(x)+(κ2−ν2)sin(x))t2αΓ(2α+1),u3(x,t)=sin(x)−(νcos⁡(x)+κsin(x))tαΓ(α+1)+(2κνcos⁡(x)+(κ2−ν2)sin(x))t2αΓ(2α+1)+((ν3−3κ2ν)cos⁡(x)+(3ν2κ−κ3)sin(x))t3αΓ(3α+1)⋮

which is the same solution as that given in [21] using the ADM.

**Example 10**

Finally, the following nonhomogeneous time FADE with variable coefficients with the initial condition is considered:

(28) ∂αu(x,t)∂tα=−xt∂2u(x,t)∂x2+t∂u(x,t)∂x+8tx2,                 t>0,  0<α≤1,u(x,0)=x2.

Using (20), the iteration formula for (28) is given by

(29) un+1(x,t)=un(x,t) −1Γ(α+1)∫0t(∂αun(x,ξ)∂ξα+xξ∂un(x,ξ)∂x−ξ∂2un(x,ξ)∂x2−2ξx2)(dξ)α.

Starting with *u*_0_(*x*, *t*) = *u*(*x*, 0) = *x* ^2^, and using (29), we have

(30) u1(x,t)=x2+8x2tα+1Γ(α+2),u2(x,t)=u3(x,t)=⋯=un(x,t)=u1(x,t).

Consequently, the exact solution is obtained as follows:

(31) u(x,t)=lim⁡n→∞un(x,t)=x2+8x2tα+1Γ(α+2).

## 5. Conclusion

The fundamental aim of this study was to obtain an analytical approximate solution of time FADEs using the FVIM. The aforementioned implementation indicates that this method is powerful and efficient in solving the equation in an easier and a more accurate way. The method also provides an analytical approximation solution in a rapidly convergent series with easily calculable terms for various physical problems. Therefore, FVIM is a more effective, a more convenient, and a more accurate method than other methods mentioned in the introduction. The obtained results denote that this method can be considered as an alternative to the other methods in the literature in terms of the purpose of solving linear or nonlinear FDEs in general.
